# Alcohol consumption among adolescents during the COVID-19 pandemic, ConVid Adolescents – Behavior Research

**DOI:** 10.1590/1980-549720230007.supl.1

**Published:** 2023-04-21

**Authors:** Deborah Carvalho Malta, Crizian Saar Gomes, Nádia Machado de Vasconcelos, Marilisa Berti de Azevedo Barros, Margareth Guimarães Lima, Paulo Roberto Borges de Souza, Celia Landmann Szwarcwald

**Affiliations:** I Universidade Federal de Minas Gerais, Nursing School, Department of Maternal-Child and Public Health Nursing – Belo Horizonte (MG), Brazil.; II Universidade Federal de Minas Gerais, Medical School, Graduate Program in Public Health – Belo Horizonte (MG), Brazil.; III Universidade Estadual de Campinas, School of Medical Sciences, Department of Collective Health – Campinas (SP), Brazil.; IV Fundação Oswaldo Cruz – Rio de Janeiro (RJ), Brazil.

**Keywords:** Underage drinking, Adolescent, COVID-19, Health surveys, Brazil

## Abstract

**Objective:**

To describe the prevalence of alcohol consumption before and during the COVID-19 pandemic and to analyze the factors associated with this behavior during the period of social distancing among Brazilian adolescents.

**Methods:**

Cross-sectional study using data from the *ConVid Adolescents* survey, carried out via the Internet between June and September 2020. The prevalence of alcohol consumption before and during the pandemic, as well as association with sociodemographic variables, mental health, and lifestyle were estimated. A logistic regression model was used to assess associated factors.

**Results:**

9,470 adolescents were evaluated. Alcohol consumption decreased from 17.70% (95%CI 16.64–18.85) before the pandemic to 12.80% (95%CI 11.85–13.76) during the pandemic. Alcohol consumption was associated with the age group of 16 and 17 years (OR=2.9; 95%CI 1.08–1.53), place of residence in the South (OR=1.82; 95%CI 1.46–2.27) and Southeast regions (OR=1.33; 95%CI 1.05–1.69), having three or more close friends (OR=1.78; 95%CI 1.25–2.53), reporting worsening sleep problems during the pandemic (OR=1.59; 95%CI 1.20–2.11), feeling sad sometimes (OR=1,83; 95%CI 1,40–2,38) and always (OR=2.27; 95%CI 1.70–3.05), feeling always irritated (OR=1,60; 95%CI 1,14–2,25), being a smoker (OR=13,74; 95%CI 8.63–21.87) and a passive smoker (OR=1.76; 95%CI 1.42–2.19). Strict adherence to social distancing was associated with lower alcohol consumption (OR=0.40; 95%CI 0.32–0.49).

**Conclusions:**

The COVID-19 pandemic led to a decrease in consumption of alcoholic beverages by Brazilian adolescents, which was influenced by sociodemographic and mental health factors, adherence to social restriction measures and lifestyle in this period. Managers, educators, family and the society must be involved in the articulation of Public Policies to prevent alcohol consumption.

## INTRODUCTION

The World Health Organization (WHO) declared the COVID-19 situation as a pandemic in March 2020^1^. To minimize the spread of the coronavirus, which causes the disease, social distancing measures were adopted in several Brazilian states and cities, with suspension of classes, closure of non-essential businesses, restrictions on travel, among others, resulting in lower social interaction^2,3^. Studies show numerous negative repercussions of measures to restrict social interaction for the health of adolescents^4–6^, such as increased symptoms of depression and feelings of anxiety, in addition to worsening of lifestyles and dissatisfaction with life. Mental suffering and feelings such as anxiety, loneliness and sadness can lead to risky consumption of alcoholic beverages and tobacco^7–9^ and, in turn, trigger substance abuse and dependence^10,11^. During the social isolation period, these substances may have been used in an attempt to alleviate unpleasant emotions^12^.

Adolescence is a period of changes and transition to adulthood, and it is common to start using psychoactive substances such as alcohol, tobacco and illicit drugs^13–15^. Drug consumption tends to increase gradually with age, which may result in dependence^16^ and exposure to immediate risks such as accidents, violence, unwanted pregnancies and sexually transmitted infections (STIs)^14,17^.

Exposure to substances in adolescence is more common in contexts of socialization such as parties, get-togethers with friends, and usually happens without the awareness and supervision of their guardians^12,16,18^. Some studies pointed out that, during the pandemic, adolescents spent more time with their parents, away from school and from friends and colleagues, which would have reduced access to these substances and, hence, their consumption^11,19–21^. However, some other studies have suggested that, in the same period, adolescents were more exposed to risk situations and managed to maintain high alcohol consumption^12,21,22^.

In Brazil, according to a study analyzing data from the *ConVid Adolescents — Research of Behaviors* survey, the consumption of alcoholic beverages among Brazilian adolescents has decreased^4^. However, studies carried out in Brazil on this topic did not assess the factors associated with this behavior. Therefore, it is important to identify the effect of social distancing on the consumption of risky substances by Brazilian adolescents and the most affected groups in order to orient public health policies and contribute to the formulation of guidelines for future periods of distancing.

The objectives of this study were to describe the prevalence of alcohol consumption by Brazilian adolescents before and during the COVID-19 pandemic and to analyze the factors associated with this behavior in the period of social distancing.

## METHODS

Cross-sectional study aiming to assess the database of the survey ConVid Adolescents — Behavior Research on virtual health, with eyes to the changes in the lives of Brazilian adolescents due to the COVID-19 pandemic.

Data were collected via the Internet, between June 27 and September 17 2020, through a self-completion questionnaire applied on a cell phone or computer. The questionnaire was built using the Research Electronic Data Capture (RedCap) application and addressed sociodemographic characteristics and changes in lifestyle, routine activities, mood and familial relationships in the period of social distancing (available at: https://convid.fiocruz.br/index.php?pag=questionario_adolescente). The information was stored on the server of the Oswaldo Cruz Foundation Institute for Scientific and Technological Communication and Information in Health (ICICT/FIOCRUZ).

Adolescents aged 12 to 17 years living in the Brazilian territory were included in the study. This age group was chosen per the definition of adolescence in the Child and Adolescent Statute^23^. Participants were invited through a chain sampling procedure called a virtual “snowball”^24^, which began with sending the questionnaire link to researchers who had experience in studies with adolescents. These researchers, in turn, sent the link to adults in their social networks who were responsible for adolescents. These adults were asked to invite at least three parents or guardians of other adolescents, so the invitations were sent to adults, who were asked: “Do you have children or are you responsible for adolescents in the age range of 12 to 17 years old?”. Only those who answered positively received the free and informed consent form with explanations about the study, a link for contact and clarifications about the research, as well as a request for consent to participate to the adolescents under their responsibility.

Upon signing of the informed consent form by an adult responsible for each adolescent, they received the free and informed assent form; only then did the participants fill out the questionnaire. Additionally, the research coordination sent letters to the directorate of state departments and schools, inviting them to send the link of the questionnaire to adolescents and their parents/guardians. The final sample consisted of 9,470 adolescents.

Since chain sampling is not probabilistic, in order to obtain a representative sample of the population, according to geographic location and sociodemographic characteristics, post-stratification procedures were used to apply weightings^25^. The sample was calibrated using data from the National School Health Survey (PeNSE, 2015) carried out by the Brazilian Institute of Geography and Statistics (IBGE) in partnership with the Ministry of Health, aiming at the same distribution by region of residence, biological sex, age group (12-15 years and 16-17 years), and school network (public or private).

Consumption of alcoholic beverages before and during the pandemic was analyzed, considering the following questions:

- “Before the pandemic, were you used to consuming alcoholic beverages at parties, get-togethers with friends, etc.?” (Yes/No);
- “During the pandemic:
  1. I did not drink alcohol;
  2. I am drinking less alcohol than I used to;
  3. I continued drinking alcohol with the same frequency;
  4. I am drinking more alcohol than I used to.”

Adolescents who answered “yes” to question A (before the pandemic) and chose options 2, 3 or 4 to answer about the pandemic period were considered consumers of alcoholic beverages.

In order to analyze the factors associated with the consumption of alcoholic beverages during the pandemic, the explanatory variables were:

1. Sociodemographic:
  - biological sex: male or female;
  - age group: 12 to 15 years and 16 and 17 years. The categorization of age groups was based on the fact that, from 16 years of age on, adolescents have more civil liability, answering personally for their actions, which can impact the decision to consume alcoholic beverages—prohibited by law until the age of 18^26^.
  - skin color: white, black, brown, other;
  - school network: private or public;
  - maternal education: primary education or less, high-school, higher education;
  - region: North, Northeast, South, Southeast and Midwest.
2. Adherence to social restriction measures:
  - Not very strict: “I didn't do anything, I had a normal life”, “I tried to be cautious, stay away from people, reduce contact a little, not visit the elderly, but I kept going out”, “I just stopped going to school, but I continued doing other activities normally”.
  - Very strict: “I stayed at home most days, going out to visit close relatives, shopping at the supermarket and pharmacy” and “I stayed strictly at home, going out only for health care needs”.
3. Mental health:
  - number of close friends: none, one friend, two friends, three or more friends;
  - quality of sleep during the pandemic: not affected, began to have sleep problems, sleep problems were maintained, sleep problems worsened, sleep problems reduced.
  - feeling sad or depressed: never/rarely, sometimes, most of the time/always.
  - feeling irritable: never/rarely, sometimes, most of the time/always.
  - feeling isolated: never/rarely, sometimes, most of the time/always.
4. Lifestyle:
  - smoking during the pandemic: yes, no;
  - passive smoker: yes, no;
  - sedentary behavior: maintained, increased, reduced. Sedentary behavior was considered to be sitting for three or more hours a day, watching television, playing video games, using a computer, cell phone, tablet or doing other activities while sitting down.
  - Practice of physical activity during the pandemic: maintained, increased, reduced. At least one hour on five or more days a week of activities was considered as physical exercise^27^.

The prevalence of alcohol consumption before and during the pandemic was estimated for the total sample and according to exposure variables, with respective 95% confidence intervals (95%CI). To verify possible factors associated with the consumption of alcoholic beverages during the pandemic, univariate and multivariate logistic regression models were used. All variables with p<0.2 in the univariate analysis were selected for the multivariate model. The significance level adopted for the multivariate analysis was 5%.

All analyses were performed using the Software for Statistics and Data Science (Stata) version 14.0 and considered post-stratification weights.

The research was approved by the National Committee for Ethics in Research (Opinion No. 4,100,515). The adolescents’ parents or guardians signed an informed consent form, followed by the assent form by the adolescents themselves, and participants were not identified.

## RESULTS

A total of 9,470 adolescents were evaluated; 50.2% (95%CI 48.6–51.9) were females, 67.7% (95%CI 66.3–69.1) were aged between 12 and 15 years old. Most of them reported being brown (46.6%; 95%CI 44.9–48.3), followed by white (40.1%; 95%CI 38.5–41.7), and studying in public schools (85.9%; 95%CI 85.1–86.7). As for maternal education, the distribution was similar, around one third in each group (Table 1).

**Table 1:** Sample characteristics (n=9,470). ConVid Adolescents — Behavior Research, 2020.

		Weighted frequency (%)	95%CI
Sex			
	Male	49.8	48.1–51.4
	Female	50.2	48.6–51.9
Age range (years)			
	12–15	67.7	66.3–69.1
	16-17	32.3	30.9–33.7
Skin color			
	White	40.1	38.5–41.7
	black	9.7	8.8–10.7
	brown	46.6	44.9–48.3
	others	3.6	3.0–4.4
School network			
	Private	14.1	13.3–14.9
	Public	85.9	85.1–86.7
Maternal education			
	elementary school or less	32.6	30.9–34.2
	High school	33.8	32.1–35.5
	Higher education	33.6	32.1–35.2

95%CI: 95% confidence interval.

**Figure 1:**
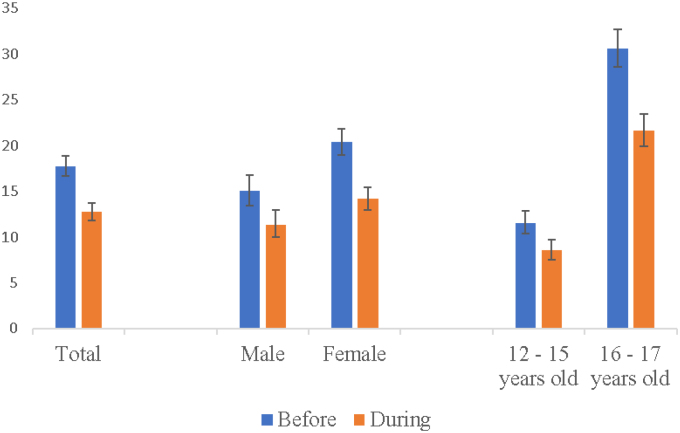
Prevalence of alcohol consumption before and during the COVID-19 pandemic. ConVid Adolescents — Behavior Research, 2020.

Consumption of alcoholic beverages was reported by 17.70% of participants (95%CI 16.64–18.85) before the pandemic, and reduced to 12.8% (95%CI 11.85–13.76) during the pandemic. There was a reduction among males (from 15.01 to 11.37%) and females (from 20.4 to 14.15%), in both cases, in the age groups analyzed: from 12 to 15 years (from 11.56 to 8.55%) and 16 and 17 years old (from 30.61 to 21.61%) (Figure 1).

As for the factors associated with the use of alcoholic beverages during social isolation, the univariate model showed a greater chance of this behavior among female adolescents (OR=1.29; 95%CI 1.08–1.53); aged 16 and 17 years (OR=2.95; 95%CI 2.48–3.51); participants living in the South (OR=1.93; 95%CI 1.59–2.35) and Southeast (OR=1.60; 95%CI 1.30–1.98) regions, compared with those living in the North Region; who had three or more close friends (OR=1.42; 95%CI 1.05–1.93); who reported starting to have sleep problems (OR=1.53; 95%CI 1.22–1.93), maintained sleep problems (OR=1.81; 95%CI 1.38–2.37) and worsened sleep problems (OR=2.78; 95%CI 2.23–3.46) during the pandemic; who reported feeling sad sometimes (OR=2.19; 95%CI 1.71–2.80) and always (OR=3.15; 95%CI 2.51–3.97); feeling irritated sometimes (OR=1.56; 95%CI 1.15–2.12) and always (OR=2.67; 95%CI 2.03–3.51); feeling isolated sometimes (OR=1.89; 95%CI 1.50–2.38) and always (OR=2.30; 95%CI 1.84–2.88); adolescents who actively smoked (OR=18.33; 95%CI 12.40–27.14) or were passive smokers (OR=2.26; 95%CI 1.86–2.74); and those who had sedentary behavior worsened (OR=1.33; 95%CI 1.11–1.59).

On the other hand, brown skin adolescents (OR=0.80; 95%CI 0.66–0.96), who studied in private schools (OR=0.70; 95%CI 0.58–0.85), whose mothers had higher education (high school — OR=0.69; 95%CI 0.56–0.86; higher education — OR=0.72; 95%CI 0.58–0.90) and who adopted social distancing measures rigorously (OR=0.46; 95%CI 0.38–0.55) were less likely to consume alcoholic beverages during the pandemic (Table 2).

**Table 2:** Factors associated with alcohol consumption by adolescents during the COVID-19 pandemic. ConVid Adolescents — Behavior Research, 2020.

Variables		% (95%CI)	OR (95%CI)	OR* (95%CI)
Sex				
	Male	11.37 (9.98–12.92)	—	
	Female	14.15 (12.99–15.40)	1.29 (1.08–1.53)	
Age range (years)				
	12–15	8.55 (7.52–9.71)	—	—
	16-17	21.61 (19.9–23.43)	2.95 (2.48–3.51)	2.90 (2.39–3.51)
Skin color				
	White	13.53 (12.19–14.99)	—	
	black	16.89 (13.70–20.65)	1.30 (0.99–1.71)	
	brown	11.12 (9.76–12.64)	0.80 (0.66–0.96)	
	others	15.12 (10.49–21.32)	1.14 (0.74–1.76)	
School network				
	Private	13.27 (12.23–14.40)	—	
	Public	9.73 (8.34–11.32)	0.70 (0.58–0.85)	
Maternal education				
	elementary school or less	15.55 (13.63–17.69)	—	
	High school	11.34 (9.93–12.92)	0.69 (0.56–0.86)	
	Higher education	11.72 (10.17–13.47)	0.72 (0.58–0.90)	
Region				
	North	9.99 (8.53–11.67)	—	—
	Northeast	7.84 (5.90–10.33)	0.77 (0.54–1.09)	
	Southeast	15.08 (13.63–16.66)	1.60 (1.30–1.98)	1.33 (1.05–1.69)
	South	17.66 (16.35–19.05)	1.93 (1.59–2.35)	1.82 (1.46–2.27)
	Midwest	13.26 (10.73–16.28)	1.38 (1.02–1.85)	
Social distancing				
	not strict	19.64 (17.48–22.00)	—	—
	very strict	10.05 (9.12–11.07)	0.46 (0.38–0.55)	0.40 (0.32–0.49)
Close friends				
	None	10.01 (7.74–12.86)	—	—
	1	11.82 (9.43–14.72)	1.20 (0.82–1.76)	
	2	12.24 (10.45–14.29)	1.25 (0.90–1.75)	
	3 or more	13.64 (12.36–15.04)	1.42 (1.05–1.93)	1.78 (1.25–2.53)
Sleep quality during the pandemic				
	not affected	9.58 (8.50–10.78)	—	—
	started having problems	13.96 (11.84–16.38)	1.53 (1.22–1.93)	
	problems maintained	16.06 (13.10–19.54)	1.81 (1.38–2.37)	
	problems got worse	22.75 (19.81–25.99)	2.78 (2.23–3.46)	1.59 (1.20–2.11)
	Problems reduced	10.88 (7.03–16.45)	1.15 (0.70–1.89)	
Feeling sad				
	Never/rarely	6.65 (5.54–7.96)	—	—
	Sometimes	13.47 (1.78–15.36)	2.19 (1.71–2.80)	1.83 (1.40–2.38)
	Most of the time/always	18.34 (16.58–20.23)	3.15 (2.51–3.97)	2.27 (1.70–3.05)
Feeling irritated				
	Never/rarely	6.97 (5.50–8.80)	—	—
	Sometimes	10.47 (8.99–12.17)	1.56 (1.15–2.12)	
	Most of the time/always	16.66 (15.21–18.21)	2.67 (2.03–3.51)	1.60 (1.14–2.25)
Feeling isolated				
	Never/rarely	8.15 (6.89–9.61)	—	
	Sometimes	14.35 (12.68–16.20)	1.89 (1.50–2.38)	
	Most of the time/always	16.93 (15.17–18.85)	2.30 (1.84–2.88)	
Smoking during the pandemic				
	No	11.36 (10.48–12.31)	—	—
	Yes	70.15 (61.6–77.48)	18.33 (12.40–27.14)	13.74 (8.63–21.87)
Passive smoker				
	No	10.66 (9.74–11.66)	—	—
	Yes	27.23 (21.24–24.15)	2.26 (1.86–2.74)	1.76 (1.42–2.19)
Sedentary behavior				
	Maintained	11.88 (10.78–13.06)	—	
	Increased	15.15 (13.38–17.11)	1.33 (1.11–1.59)	
	Reduced	12.12 (7.54–18.91)	1.02 (0.60–1.75)	
practice of physical activity				
	Maintained	12.31 (11.23–13.48)	—	
	Increased	14.47 (12.46–16.74)	1.20 (0.98–1.47)	
	Reduced	12.99 (10.02–16.66)	1.06 (0.78–1.45)	

OR: *odds ratio*; 95% CI: 95% confidence interval;

* multivariate model.

In the multivariate model, the following variables were associated with higher consumption of alcoholic beverages: age group of 16 and 17 years (OR=2.90; 95%CI 2.39–3.51); living in the South (OR=1.82; 95%CI 1.46–2.27) and Southeast regions (OR=1.33; 95%CI 1.05–1.69); having three or more close friends (OR=1.78; 95%CI 1.25–2.53); worsened sleep problems during the pandemic (OR=1.59; 95%CI 1.20–2.11); feeling sad sometimes (OR=1.83; 95%CI 1.40–2.38) and always (OR=2.27; 95%CI 1.77–3.12); always feeling irritable (OR=1.60; 95%CI 1.14–2.25); and being an active (OR=13.74; 95%CI 8.63–21.87) or passive smoker (OR=1.76; 95%CI 1.42–2.19). Meanwhile, lower consumption of alcoholic beverages was associated with very strict adherence to social distancing measures (OR=0.40; 95%CI 0.32–0.49) (Table 2).

## DISCUSSION

This study detected a decrease in alcohol consumption during the pandemic, which was reported by about a fifth of the participating adolescents. Regarding those who consumed alcoholic beverages in the period, the highest consumption was among older adolescents (16 and 17 years old) who lived in the South and Southeast regions, with three or more close friends, who reported worse sleep and feelings of sadness and irritability, and reported being active and passive smokers. Consumption was lower by adolescents who took very strict social distancing measures during the pandemic.

The reduction in prevalence of alcohol consumption during the pandemic, observed in the present study is in line with other studies^12,28^. It may be related to numerous factors, mainly less opportunities to participate in parties, celebrations and get-togethers with friends due to the guidelines for social distancing^12^. PeNSE data showed that the main opportunities to obtain alcoholic beverages are parties (29.2%) and other events in the company of friends (17.7%)^29^.

Despite this reduction, the high prevalence of alcohol consumption during the pandemic (12.7%) disturbingly shows the continuity of this behavior among adolescents, even during social distancing. Other studies have already reported that alcohol consumption is high among Brazilian adolescents^19,30^. According to PeNSE 2019, 63.3% of students aged 13 to 17 years old at least tried these products and 25%^29^ had consumed them in the last 30 days. Similar to Brazil, alcoholic beverages are the drug most consumed by young people in other countries^13,17,31,32^.

The results also pointed to complex relationships between individual and contextual factors, behavioral changes and the use of risky substances by adolescents during the COVID-19 confinement.

Alcohol consumption during the pandemic was almost three times higher in older adolescents. These data are consistent with the findings of PeNSE, according to which adolescents aged 16 and 17 years consume more alcoholic beverages than those aged 13 to 15 years^29^. Despite the closure of leisure establishments and the reduction in social gatherings during the period studied, the older group maintained more freedom to leave the house and, therefore, more opportunities to access psychoactive substances^19,33^. In addition, some parents approved the consumption of alcoholic beverages by older adolescents at home, usually not allowed before^34^.

Adolescents from the South and Southeast regions had a higher consumption of alcoholic beverages during the pandemic than those living in the North region of the country. This result accompanies the pre-pandemic national prevalence, since adolescents from these regions had a higher rate of trying and consuming alcoholic beverages, as well as more episodes of drunkenness^29^.

Adolescents with three close friends or more drank about three times more, which makes it an important marker of use. This indicator reflects the influence of peers on this habit. Previous studies have shown the influence of friends on alcohol consumption, as adolescents with more friends tend to be more popular, which gives them more access to parties, where there is a higher consumption of alcoholic beverages^18^. On the other hand, strict social distancing, which reduced the presence of this group at parties and contact with friends, resulted in a drop in alcohol consumption, also described by other studies^12,19,35^.

Our study also identified that alcohol consumption was higher among adolescents who reported symptoms of mental health worsening, such as feelings of sadness, irritation and poor sleep quality. The pandemic meant, for most adolescents, distancing from friends and a change in routine, as many remained alone at home, which may have influenced the increase in feelings of sadness and irritability, and worsening of sleep quality^4,6^. In this setting, some adolescents may have used risky substances as a way of dealing with bad feelings in the social distancing phase^12,21,36,37^. In addition, being home alone may have facilitated access to alcoholic beverages, since many guardians had to be away from home to work, which reduced the control and supervision of the underages^12^.

Another factor that was associated with the consumption of alcoholic beverages was the habit of smoking. Studies indicate that tobacco can trigger other risk behaviors, such as drinking^38,39^, with an increase in the feeling of pleasure due to the combination of nicotine and alcohol^40^. Adolescents who reported being passive smokers at home were also more likely to consume alcoholic beverages. Studies show that passive smokers at home are a more vulnerable population^41^, which may be a marker of homes in which adults supervise and protect less adolescents or even encourage the consumption of alcoholic beverages at home. As reported by PeNSE, approximately 11% of students who consumed alcoholic beverages were given access to the product by someone in their own family^29^, which also shows how the consumption of alcoholic beverages is naturalized in Brazilian culture.

Adolescents who adhered strictly to social distancing measures were less likely to consume alcoholic beverages during the pandemic, which may be related to distancing from friends and parties and, consequently, difficulty in accessing them, and better monitoring by parents or guardians about this consumption^12,36^.

This is, to date, the first nationally representative study that analyzed factors associated with alcohol consumption by adolescents during the COVID-19 pandemic. However, some limitations need to be mentioned. The sample selected via the Internet, not random, may not have reached all social segments, but sample calibration based on PeNSE data reduced this limitation. Data were collected at a specific time of the pandemic; nowadays, the scenario may be different. Therefore, further studies on other periods of the pandemic are recommended.

As evidenced by the results of this study, the COVID-19 pandemic affected the social life of young people and reduced the consumption of alcoholic beverages by Brazilian adolescents, a phenomenon associated with sociodemographic factors (age and region of residence), adherence to social distancing measures social, events related to mental health (number of friends, quality of sleep and feelings of sadness and irritability) and lifestyle (active and passive smoking and sedentary behavior).

It all evidence that public policies for health promotion and alcohol consumption prevention must be articulated and involve authorities, educators, family and the society. It is urgent to involve the society in the debate on the consumption of alcoholic beverages by adolescents, establishing conditions to advance in the legislation, with better regulation from the supply to the sale of alcoholic beverages, thinking mainly about the prohibition of marketing of these substances, just as there are rules for the sale of cigarettes.
