# Supplementary material for: Alcohol consumption among adolescents during the COVID-19 pandemic, ConVid Adolescents – Behavior Research
Source: Rev Bras Epidemiol. 2023 Apr 21;26(Suppl 1):e230007. doi: 10.1590/1980-549720230007.supl.1 (PMC10176733; doi:10.1590/1980-549720230007.supl.1)
Supplement: Translation

# Translation: O consumo de bebidas alcoólicas entre adolescentes durante a pandemia de COVID-19, ConVid Adolescentes — Pesquisa de Comportamentos

**DOI:** 10.1590/1980-549720230007.supl.1.1

**Authors:** Deborah Carvalho Malta, Crizian Saar Gomes, Nádia Machado de Vasconcelos, Marilisa Berti de Azevedo Barros, Margareth Guimarães Lima, Paulo Roberto Borges de Souza, Celia Landmann Szwarcwald

**Affiliations:** I Universidade Federal de Minas Gerais, Escola de Enfermagem, Departamento de Enfermagem Materno Infantil e Saúde Pública – Belo Horizonte (MG), Brasil.; II Universidade Federal de Minas Gerais, Faculdade de Medicina, Programa de Pós-Graduação em Saúde Pública – Belo Horizonte (MG), Brasil.; II Universidade Estadual de Campinas, Faculdade de Ciências Médicas, Departamento de Saúde Coletiva – Campinas (SP), Brasil.; IV Fundação Oswaldo Cruz – Rio de Janeiro (RJ), Brasil.

**Keywords:** Consumo de bebidas alcoólicas por menores, Adolescentes, COVID-19, Inquéritos epidemiológicos, Brasil

## Abstract

**Objetivo:**

Descrever as prevalências do consumo de bebidas alcoólicas por adolescentes brasileiros antes e durante a pandemia de COVID-19 e analisar os fatores associados a esse comportamento no período de distanciamento social.

**Métodos:**

Estudo transversal, utilizando dados da pesquisa ConVid Adolescentes, realizado via web entre junho e setembro de 2020. Foi estimada a prevalência do consumo de bebidas alcoólicas antes e durante a pandemia e a associação com variáveis sociodemográficas, de saúde mental e estilos de vida. Foi usado modelo de regressão logística para avaliar os fatores associados.

**Resultados:**

Avaliaram-se 9.470 adolescentes. O consumo de bebida alcoólica reduziu de 17,70% (IC95% 16,64–18,85), antes da pandemia, para 12,80% (IC95% 11,85–13,76), durante a pandemia. O consumo de bebidas alcoólicas esteve associado à faixa etária de 16 e 17 anos (OR=2,9; IC95% 1,08–1,53), morar na Região Sul (OR=1,82; IC95% 1,46–2,27) e Sudeste (OR=1,33; IC95% 1,05–1,69), ter três ou mais amigos próximos (OR=1,78; IC95% 1,25–2,53), relatar piora dos problemas de sono (OR=1,59; IC95% 1,20–2,11), sentir-se triste às vezes (OR=1,83; IC95% 1,40–2,38) e sempre (OR=2,27; IC95% 1,70–3,05), irritado sempre (OR=1,60; IC95% 1,14–2,25), ser fumante ativo (OR=13,74; IC95% 8,63–21,87) e fumante passivo (OR=1,76; IC95% 1,42–2,19). A adesão à restrição de forma muito rigorosa associou-se ao menor consumo de bebidas alcoólicas (OR=0,40; IC95% 0,32–0,49).

**Conclusão:**

A pandemia causada pela COVID-19 levou à diminuição no consumo de bebidas alcoólicas pelos adolescentes brasileiros, e o consumo durante a pandemia foi influenciado por fatores sociodemográficos, de saúde mental, adesão às medidas de restrição social e estilos de vida. Faz-se necessário o envolvimento de gestores, educadores, família e sociedade na articulação de políticas públicas para evitar o consumo de bebidas alcoólicas.

## INTRODUÇÃO

A Organização Mundial da Saúde (OMS) declarou situação de pandemia de COVID-19 em março de 2020^1^. Visando minimizar a disseminação do coronavírus, causador da doença, foram adotadas medidas de distanciamento social em vários estados e cidades brasileiros, com a suspensão das aulas, fechamento do comércio não essencial, restrição dos deslocamentos, dentre outras, o que resultou em redução do convívio social^2,3^. Estudos evidenciam inúmeras repercussões negativas para a saúde dos adolescentes decorrentes das medidas de restrição do convívio social^4–6^, como aumento de sintomas de depressão e sentimentos de ansiedade, além de piora nos estilos de vida e satisfação com a vida. O sofrimento mental e os sentimentos, como ansiedade, solidão e tristeza, podem levar ao consumo de risco de bebidas alcoólicas e tabaco^7–9^, o que pode desencadear o abuso e a dependência dessas susbstâncias^10,11^. No período de isolamento social, o consumo dessas substâncias pode ter sido utilizado na busca de aliviar as emoções desagradáveis^12^.

A adolescência é um período de mudanças e transição para a idade adulta, em que é comum a iniciação do uso de substâncias psicoativas, como álcool, tabaco e drogas ilícitas^13–15^. O consumo de drogas tende a aumentar gradativamente com a idade, podendo resultar em dependência^16^ e na exposição a riscos imediatos, como acidentes, violências, gravidez indesejada e infecções sexualmente transmissíveis (ISTs)^14,17^.

A exposição a substâncias na adolescência é mais frequente nos contextos de socialização com os pares, como festas, encontros com amigos, e acontece, em geral, sem o conhecimento e a supervisão dos responsáveis^12,16,18^. Alguns estudos apontaram que, durante a pandemia, os adolescentes passaram mais tempo com os pais, longe da escola e dos amigos e colegas, o que teria reduzido o acesso a essas substâncias, reduzindo o consumo^11,19–21^. Entretanto, outros estudos sugeriram que, no mesmo período, os adolescentes tiveram maior exposição a situações de risco e conseguiram manter o consumo de bebidas alcoólicas elevado^12,21,22^.

No Brasil, conforme estudo analisando dados da pesquisa ConVid Adolescentes — Pesquisa de Comportamentos, diminuiu o consumo de bebidas alcoólicas entre adolescentes brasileiros^4^. Contudo, os estudos realizados no Brasil sobre essa temática não analisaram os fatores associados a esse comportamento. Nesse sentido, é importante identificar o efeito do distanciamento social no consumo de substâncias de risco por adolescentes brasileiros e os grupos mais afetados a fim de direcionar políticas de saúde pública e contribuir para a formulação de orientações para futuros períodos de distanciamento.

Os objetivos deste estudo eram descrever as prevalências de consumo de bebidas alcoólicas por adolescentes brasileiros antes e durante a pandemia de COVID-19 e analisar os fatores associados a esse comportamento durante o período de distanciamento social.

## MÉTODOS

Estudo transversal, que analisa a base de dados da pesquisa ConVid Adolescentes — Pesquisa de Comportamentos, inquérito sobre saúde virtual, visando avaliar as mudanças na vida dos adolescentes brasileiros em função da pandemia de COVID-19.

Os dados foram coletados via *web*, entre 27 de junho e 17 de setembro de 2020, por meio de um questionário de autopreenchimento, por meio de celular ou computador. O questionário foi construído por meio do aplicativo Research Eletronic Data Capture (RedCap) e abordou questões sobre as características sociodemográficas e as mudanças nos estilos de vida, nas atividades de rotina, no estado de ânimo e nas relações familiares no período de distanciamento social (disponível em: https://convid.fiocruz.br/index.php?pag=questionario_adolescente). As informações foram armazenadas no servidor do Instituto de Comunicação e Informação Científica e Tecnológica em Saúde da Fundação Oswaldo Cruz (ICICT/FIOCRUZ).

Foram incluídos no estudo os adolescentes entre 12 e 17 anos do território brasileiro. Essa faixa etária foi escolhida segundo a definição de adolescência do Estatuto da Criança e do Adolescente^23^. O convite aos participantes foi feito por um procedimento de amostragem em cadeia chamado “bola de neve” virtual^24^, que se iniciou com o envio do *link* do questionário para pesquisadores que tivessem experiência em estudos com adolescentes. Esses pesquisadores, por sua vez, enviaram o *link* para adultos das suas redes sociais que fossem responsáveis por adolescentes. A esses adultos, foi solicitado convidar pelo menos três pais ou responsáveis por adolescentes, assim os convites foram enviados aos adultos, aos quais se perguntou: “O(a) Sr.(a) tem filhos ou é responsável por jovens na faixa de idade de 12 a 17 anos?” Somente aqueles que responderam afirmativamente receberam o Termo de Consentimento Livre e Esclarecido (TCLE) com explicações sobre o estudo, um *link* para contato e esclarecimentos sobre a pesquisa, bem como solicitação de consentimento de participação do menor sob sua responsabilidade.

Mediante a aceitação do TCLE pelo adulto responsável, o adolescente recebeu o Termo de Assentimento Livre e Esclarecido (TALE) e, em seguida, o respondente preencheu o questionário. Além disso, a coordenação da pesquisa enviou cartas para a direção de secretarias estaduais e de escolas, convidando-as para enviar o *link* para os pais e os adolescentes. A amostra obtida foi de 9.470 adolescentes.

Uma vez que a amostragem por redes não é probabilística, para obter uma amostra representativa da população, de acordo com a localização geográfica e as características sociodemográficas, foram realizadas ponderações calculadas por procedimentos de pós-estratificação^25^. A amostra foi calibrada por meio dos dados da Pesquisa Nacional de Saúde Escolar (PeNSE, 2015) do Instituto Brasileiro de Geografia e Estatística (IBGE), em parceria com o Ministério da Saúde, visando à mesma distribuição por região de residência, sexo, faixa etária (12 a 15 anos e 16 e 17 anos) e tipo de escola (pública ou privada).

No presente estudo, foi analisado o consumo de bebidas alcoólicas antes e durante a pandemia, considerando as seguintes perguntas:

- “Antes da pandemia, você costumava consumir bebidas alcoólicas em festas, saídas com amigos, etc.?” (Sim/Não);
- “Durante a pandemia:
  1. Não tomei bebida alcoólica;
  2. Estou tomando menos bebida alcoólica do que costumava;
  3. Continuei tomando bebida alcoólica com a mesma frequência;
  4. Estou tomando mais bebida alcoólica do que costumava”. Foram considerados como consumidores de bebidas alcoólicas os adolescentes que responderam “sim” para a questão A (antes da pandemia) e para as opções 2, 3 ou 4, durante a pandemia.

Visando analisar os fatores associados ao consumo de bebidas alcoólicas durante a pandemia, as variáveis explicativas investigadas foram:

1. Sociodemográficas:
  - Sexo: masculino ou feminino;
  - Faixa etária: 12 a 15 anos e 16 e 17 anos. A categorização das faixas etárias foi baseada no fato de os adolescentes, a partir de 16 anos, já apresentarem mais responsabilidade civil, respondendo pessoalmente por seus atos, o que pode impactar a decisão de consumir bebidas alcoólicas, o que em princípio está proibido até os 18 anos^26^.
  - Raça/cor: branca, preta, parda, outras;
  - Tipo de escola: privada ou pública;
  - Escolaridade materna: ensino fundamental ou menos, ensino médio, ensino superior;
  - Região: Norte, Nordeste, Sul, Sudeste e Centro-Oeste.
2. Adesão às medidas de restrição social:
  - Pouco rigorosa: “Não fiz nada, levei vida normal”, “Procurei tomar cuidados, ficar a distância das pessoas, reduzir um pouco o contato, não visitar idosos, mas continuei saindo”, “Somente deixei de ir à escola, mas segui normalmente com outras atividades”.
  - Muito rigorosa: “Fiquei em casa na maior parte dos dias, saindo para casa de familiares próximos, compras em supermercado e farmácia” e “Fiquei rigorosamente em casa, saindo só por necessidades de atendimento à saúde”.
3. Saúde mental:
  - Número de amigos próximos: nenhum, um amigo, dois amigos, três ou mais amigos;
  - Qualidade do sono durante a pandemia: não afetou, começou a ter problemas de sono, problemas de sono foram mantidos, problemas de sono pioraram, problemas de sono reduziram.
  - Sentir-se triste ou deprimido: nunca/raramente, às vezes, maioria das vezes/sempre.
  - Sentir-se irritado: nunca/raramente, às vezes, maioria das vezes/sempre.
  - Sentir-se isolado: nunca/raramente, às vezes, maioria das vezes/sempre.
4. Estilos de vida:
  - Hábito de fumar durante a pandemia: sim, não;
  - Fumante passivo: sim, não;
  - Comportamento sedentário: manteve, aumentou, reduziu. Considerou-se comportamento sedentário ficar três ou mais horas por dia sentado, assistindo à televisão, jogando *videogame*, usando computador, celular, *tablet* ou fazendo outras atividades sentado.
  - Prática de atividade física durante a pandemia: manteve, aumentou, reduziu. Considerou-se praticar exercício físico pelo menos uma hora em cinco ou mais dias por semana^27^.

Foram estimadas as prevalências do consumo de bebidas alcoólicas antes e durante a pandemia para amostra total e segundo variáveis de exposição, com os respectivos intervalos de confiança de 95% (IC95%). Para verificar os possíveis fatores associados ao consumo de bebidas alcoólicas durante a pandemia, foi utilizado o modelo de regressão logística uni e multivariado. Todas as variáveis com p < 0,2 na análise univariada foram selecionadas para o modelo multivariado. O nível de significância adotado no modelo multivariado foi de 5%.

Todas as análises foram realizadas no *Software for Statistics and Data Science* (*Stata)* versão 14.0 e consideraram os pesos pós-estratificação.

A pesquisa foi aprovada pela Comissão Nacional de Ética em Pesquisa (Parecer n^o^ 4.100.515). Os pais dos adolescentes ou seus responsáveis preencheram previamente um TCLE seguido do assentimento dos próprios adolescentes, os quais não foram identificados.

## RESULTADOS

Foram avaliados 9.470 adolescentes, sendo 50,2% (IC95% 48,6–51,9) do sexo feminino e 67,7% (IC95% 66,3–69,1) entre 12 e 15 anos. A maioria declarou ser de cor da pele parda (46,6%; IC95% 44,9–48,3), seguida de branca (40,1%; IC95% 38,5–41,7) e estudar em escolas públicas (85,9%; IC95% 85,1–86,7). Quanto à escolaridade materna, a distribuição foi semelhante, cerca de um terço em cada grupo (Tabela 1).

**Tabela 1:** Características da amostra (n=9.470). ConVid Adolescentes — Pesquisa de Comportamentos, 2020.

		Frequência ponderada (%)	IC95%
Sexo			
	Masculino	49,8	48,1–51,4
	Feminino	50,2	48,6–51,9
Faixa etária (anos)			
	12–15	67,7	66,3–69,1
	16 e 17	32,3	30,9–33,7
Raça/cor da pele			
	Branca	40,1	38,5–41,7
	Preta	9,7	8,8–10,7
	Parda	46,6	44,9–48,3
	Outras	3,6	3,0–4,4
Tipo de escola			
	Privada	14,1	13,3–14,9
	Pública	85,9	85,1–86,7
Escolaridade materna			
	Ensino fundamental ou menos	32,6	30,9–34,2
	Ensino médio	33,8	32,1–35,5
	Ensino superior	33,6	32,1–35,2

IC: intervalo de confiança de 95%.

O consumo de bebidas alcoólicas foi relatado por 17,70% (IC95% 16,64–18,85) antes da pandemia e reduziu para 12,8% (IC95% 11,85–13,76) durante a pandemia. Houve redução tanto para os meninos (de 15,01 para 11,37%) quanto para as meninas (de 20,4 para 14,15%), em ambos os casos, nas faixas etárias analisadas: de 12 a 15 anos (de 11,56 para 8,55%) e de 16 e 17 anos (de 30,61 para 21,61%) (Figura 1).

**Figura 1:** Prevalência do consumo de bebidas alcoólicas antes e durante a pandemia de COVID-19. ConVid Adolescentes — Pesquisa de Comportamentos, 2020.

Quanto aos fatores associados ao uso de bebidas alcoólicas durante o isolamento social, verificou-se, no modelo univariado, maior chance desse comportamento nos adolescentes do sexo feminino (OR=1,29; IC95% 1,08–1,53); de 16 e 17 anos (OR=2,95; IC95% 2,48–3,51); nos que moram nas regiões Sul (OR=1,93; IC95% 1,59–2,35) e Sudeste (OR=1,60; IC95% 1,30–1,98), se comparados com os da Região Norte; os que tinham três ou mais amigos próximos (OR=1,42; IC95% 1,05–1,93); que relataram que os problemas de sono iniciaram (OR=1,53; IC95% 1,22–1,93), foram mantidos tais distúrbios (OR=1,81; IC95% 1,38–2,37) e pioraram (OR=2,78; IC95% 2,23–3,46) durante a pandemia; que relataram sentir-se triste às vezes (OR=2,19; IC95% 1,71–2,80) e sempre (OR=3,15; IC95% 2,51–3,97); sentir-se irritado às vezes (OR=1,56; IC95% 1,15–2,12) e sempre (OR=2,67; IC95% 2,03–3,51); sentir-se isolado às vezes (OR=1,89; IC95% 1,50–2,38) e sempre (OR=2,30; IC95% 1,84–2,88); nos adolescentes que fumavam ativamente (OR=18,33; IC95% 12,40–27,14) ou eram fumantes passivos (OR=2,26; IC95% 1,86–2,74); e nos que aumentaram o comportamento sedentário (OR=1,33; IC95% 1,11–1,59).

Por outro lado, adolescentes de cor parda (OR=0,80; IC95% 0,66–0,96), que estudavam em escolas privadas (OR=0,70; IC95% 0,58–0,85), cujas mães tinham maior escolaridade (ensino médio — OR=0,69; IC95% 0,56–0,86; ensino superior — OR=0,72; IC95% 0,58–0,90) e que adotaram as medidas de distanciamento de forma rigorosa (OR=0,46; IC95% 0,38–0,55) tiveram menor chance de consumir bebidas alcoólicas durante a pandemia (Tabela 2).

**Tabela 2:** Fatores associados ao consumo de bebidas alcoólicas por adolescentes durante a pandemia de COVID-19. ConVid Adolescentes — Pesquisa de Comportamentos, 2020.

Variáveis		% (IC95%)	OR (IC95%)	OR* (IC95%)
Sexo				
	Masculino	11,37 (9,98–12,92)	—	
	Feminino	14,15 (12,99–15,40)	1,29 (1,08–1,53)	
Faixa etária (anos)				
	12 a 15	8,55 (7,52–9,71)	—	—
	16 e 17	21,61 (19,9–23,43)	2,95 (2,48–3,51)	2,90 (2,39–3,51)
Raça/cor da pele				
	Branca	13,53 (12,19–14,99)	—	
	Preta	16,89 (13,70–20,65)	1,30 (0,99–1,71)	
	Parda	11,12 (9,76–12,64)	0,80 (0,66–0,96)	
	Outras	15,12 (10,49–21,32)	1,14 (0,74–1,76)	
Tipo de escola				
	Pública	13,27 (12,23–14,40)	—	
	Privada	9,73 (8,34–11,32)	0,70 (0,58–0,85)	
Escolaridade materna				
	Ensino fundamental ou menos	15,55 (13,63–17,69)	—	
	Ensino médio	11,34 (9,93–12,92)	0,69 (0,56–0,86)	
	Ensino superior	11,72 (10,17–13,47)	0,72 (0,58–0,90)	
Região				
	Norte	9,99 (8,53–11,67)	—	—
	Nordeste	7,84 (5,90–10,33)	0,77 (0,54–1,09)	
	Sudeste	15,08 (13,63–16,66)	1,60 (1,30–1,98)	1,33 (1,05–1,69)
	Sul	17,66 (16,35–19,05)	1,93 (1,59–2,35)	1,82 (1,46–2,27)
	Centro-Oeste	13,26 (10,73–16,28)	1,38 (1,02–1,85)	
Restrição social				
	Pouco rigorosa	19,64 (17,48–22,00)	—	—
	Muito rigorosa	10,05 (9,12–11,07)	0,46 (0,38–0,55)	0,40 (0,32–0,49)
Amigos próximos				
	Nenhum	10,01 (7,74–12,86)	—	—
	1	11,82 (9,43–14,72)	1,20 (0,82–1,76)	
	2	12,24 (10,45–14,29)	1,25 (0,90–1,75)	
	3 ou mais	13,64 (12,36–15,04)	1,42 (1,05–1,93)	1,78 (1,25–2,53)
Qualidade do sono durante a pandemia				
	Não afetou	9,58 (8,50–10,78)	—	—
	Começou a ter problemas	13,96 (11,84–16,38)	1,53 (1,22–1,93)	
	Problemas mantidos	16,06 (13,10–19,54)	1,81 (1,38–2,37)	
	Problemas pioraram	22,75 (19,81–25,99)	2,78 (2,23–3,46)	1,59 (1,20–2,11)
	Problemas reduziram	10,88 (7,03–16,45)	1,15 (0,70–1,89)	
Sentir-se triste				
	Nunca/raramente	6,65 (5,54–7,96)	—	—
	Às vezes	13,47 (1,78–15,36)	2,19 (1,71–2,80)	1,83 (1,40–2,38)
	Maioria das vezes/sempre	18,34 (16,58–20,23)	3,15 (2,51–3,97)	2,27 (1,70–3,05)
Sentir-se irritado				
	Nunca/raramente	6,97 (5,50–8,80)	—	—
	Às vezes	10,47 (8,99–12,17)	1,56 (1,15–2,12)	
	Maioria das vezes/sempre	16,66 (15,21–18,21)	2,67 (2,03–3,51)	1,60 (1,14–2,25)
Sentir-se isolado				
	Nunca/raramente	8,15 (6,89–9,61)	—	
	Às vezes	14,35 (12,68–16,20)	1,89 (1,50–2,38)	
	Maioria das vezes/sempre	16,93 (15,17–18,85)	2,30 (1,84–2,88)	
Hábito de fumar durante a pandemia				
	Não	11,36 (10,48–12,31)	—	—
	Sim	70,15 (61,6–77,48)	18,33 (12,40–27,14)	13,74 (8,63–21,87)
Fumante passivo				
	Não	10,66 (9,74–11,66)	—	—
	Sim	27,23 (21,24–24,15)	2,26 (1,86–2,74)	1,76 (1,42–2,19)
Comportamento sedentário				
	Manteve	11,88 (10,78–13,06)	—	
	Aumentou	15,15 (13,38–17,11)	1,33 (1,11–1,59)	
	Reduziu	12,12 (7,54–18,91)	1,02 (0,60–1,75)	
Prática de atividade física				
	Manteve	12,31 (11,23–13,48)	—	
	Aumentou	14,47 (12,46–16,74)	1,20 (0,98–1,47)	
	Reduziu	12,99 (10,02–16,66)	1,06 (0,78–1,45)	

OR: *odds ratio*; IC95%: intervalo de confiança de 95%;

* modelo multivariado.

No modelo multivariado, as seguintes variáveis associaram-se ao maior consumo de bebidas alcoólicas: faixa etária de 16 e 17 anos (OR=2,90; IC95% 2,39–3,51); morar nas regiões Sul (OR=1,82; IC95% 1,46–2,27) e Sudeste (OR=1,33; IC95% 1,05–1,69); ter três ou mais amigos próximos (OR=1,78; IC95% 1,25–2,53); os problemas de sono pioraram (OR=1,59; IC95% 1,20–2,11) durante a pandemia; sentir-se triste às vezes (OR=1,83; IC95% 1,40–2,38) e sempre (OR=2,27; IC95% 1,77–3,12); sentir-se irritado sempre (OR=1,60; IC95% 1,14–2,25); e ser fumante ativo (OR=13,74; IC95% 8,63–21,87) ou passivo (OR=1,76; IC95% 1,42–2,19). Enquanto isso, o menor consumo de bebidas alcoólicas associou-se com a adesãoo à restrição de forma muito rigorosa (OR=0,40; IC95% 0,32–0,49) (Tabela 2).

## DISCUSSÃO

O presente estudo detectou diminuição do consumo de bebidas alcoólicas durante a pandemia, o que foi referido por cerca de um quinto dos adolescentes consultados. Em relação aos que usaram bebidas alcoólicas no período, o maior consumo esteve associado aos adolescentes mais velhos (16 e 17 anos), que residiam nas regiões Sul e Sudeste, com três ou mais amigos, que referiram piora do sono e sentimento de tristeza e irritabilidade, e relataram ser fumantes ativos e passivos. O uso foi menor nos adolescentes que adotaram medidas muito rigorosas de distanciamento social na pandemia.

A redução da prevalência de consumo de bebidas alcóolicas durante a pandemia, observada no presente estudo, está em consonância com outras pesquisas^12,28^. A redução no seu consumo pode estar relacionada a inúmeros fatores, em especial, à menor oportunidade de participar de festas, celebrações e encontros com amigos em função das orientações de distanciamento social^12^. Dados da PeNSE mostraram que as principais oportunidades para obter bebidas alcoólicas são as festas (29,2%) e outros momentos na companhia dos amigos (17,7%)^29^.

Apesar dessa redução, a elevada prevalência de consumo de bebidas alcóolicas durante a pandemia (12,7%) mostra de forma preocupante a continuidade desse comportamento entre adolescentes, mesmo com o distanciamento social. Outras pesquisas já observaram que o consumo de bebidas alcoólicas é elevado entre adolescentes brasileiros^19,30^. De acordo com a PeNSE 2019, 63,3% dos escolares de 13 a 17 anos experimentaram esses produtos e 25%^29^ os consumiram nos últimos 30 dias de. De forma semelhante à do Brasil, as bebidas alcoólicas também são a droga mais consumida pelos jovens de outros países^13,17,31,32^.

Os resultados do estudo ainda apontaram complexas relações entre os fatores individuais, contextuais e mudanças comportamentais e o uso de substâncias de risco por adolescentes durante o confinamento provocado pela COVID-19.

O consumo de bebidas alcoólicas durante a pandemia foi quase três vezes mais elevado nos adolescentes mais velhos. Esse dado é concordante com os achados da PeNSE, segundo os quais adolescentes de 16 e 17 anos consomem mais bebidas alcoólicas do que os da faixa de 13 a 15 anos^29^. Apesar do fechamento dos estabelecimentos de lazer e da redução das reuniões sociais no período estudado, o grupo mais velho manteve mais liberdade para sair de casa e, com isso, mais oportunidades de acesso a substâncias psicoativas^19,33^. Além disso, alguns pais liberaram o consumo de bebidas alcoólicas pelos adolescentes mais velhos dentro de casa, o que antes não era permitido^34^.

Os adolescentes das regiões Sul e Sudeste apresentaram maior consumo de bebidas alcoólicas durante a pandemia que aqueles residentes na Região Norte do país. Esse resultado acompanha as prevalências nacionais pré-pandêmicas, visto que adolescentes dessas regiões apresentaram mais alto índice de experimentação e consumo de bebidas alcoólicas, bem como episódios de embriaguez^29^.

Os adolescentes com três amigos ou mais beberam cerca de três vezes mais, constituindo um importante marcador do uso. Esse indicador reflete a influência dos pares nesse hábito. Estudos anteriores mostraram a influência de amigos no consumo de bebidas alcoólicas, pois adolescentes com mais amigos tendem a ser mais populares, o que lhes dá mais acesso a festas, onde há maior consumo de bebidas alcoólicas^18^. Por outro lado, estar em distanciamento social rigoroso, o que reduziu a presença desse público em festas, bem como o contato com amigos, resultou na queda do consumo de bebidas alcoólicas, o que também foi descrito em outros estudos^12,19,35^.

O estudo também identificou que o consumo de bebidas alcoólicas foi maior entre adolescentes que referiram sintomas de piora da saúde mental, como sentimentos de tristeza, irritação e piora na qualidade do sono. A pandemia significou, para a maioria dos adolescentes, afastamento dos amigos e mudança na rotina, pois muitos permaneceram sozinhos em casa, o que pode ter influenciado o aumento da tristeza, da irritabilidade e a piora na qualidade do sono^4,6^. Nesse cenário, alguns adolescentes podem ter usado substâncias de risco como forma de lidar com esses sentimentos ruins na fase de distanciamento social^12,21,36,37^. Além disso, estar em casa sozinho pode ter facilitado o acesso a bebidas alcoólicas, uma vez que muitos responsáveis tiveram que se ausentar do lar para trabalhar, o que reduziu o controle e a supervisão dos menores^12^.

Outro fator que se mostrou associado ao consumo de bebidas alcoólicas foi o hábito de fumar. Estudos indicam que o tabaco pode desencadear a adoção de outros comportamentos de risco, como o consumo de bebidas^38,39^, com intensificação da sensação de prazer em decorrência da combinação de nicotina e álcool^40^. Os adolescentes que reportaram ser fumantes passivos no domicílio também tiveram mais chance de consumir bebidas alcoólicas. Estudos evidenciam que os fumantes passivos no domicílio representam uma população mais vulnerável^41^, o que pode ser um marcador de lares nos quais os adultos exercem menor supervisão e proteção dos adolescentes ou até mesmo estimulam o consumo de bebidas alcoólicas no domicílio. Como constatou a PeNSE, os cerca de 11% dos escolares que consumiam bebidas alcoólicas obtiveram o produto com alguém da própria família^29^, o que também mostra quão naturalizado é o consumo de bebidas alcoólicas na cultura brasileira.

Os adolescentes que aderiram de forma severa às medidas de restrição social tiveram menos chances de consumir bebidas alcoólicas durante a pandemia, o que pode estar relacionado ao distanciamento dos amigos e das festas e consequentemente à maior dificuldade de acesso, além de haver melhor controle por parte dos pais ou responsáveis sobre esse consumo^12,36^.

Este é, até o momento, o primeiro estudo nacionalmente representativo a analisar os fatores associados ao consumo de bebidas alcoólicas por adolescentes durante a pandemia de COVID-19. No entanto, algumas limitações deste estudo precisam ser mencionadas. A amostra selecionada via *web*, não aleatória, pode não ter alcançado todos os segmentos sociais, no entanto, a calibração da amostra baseada em dados da PeNSE reduziu essa limitação. Os dados foram coletados em um tempo específico da pandemia; hoje o cenário pode estar diferente. Assim se recomendam estudos em outros períodos da pandemia.

Como evidenciam os resultados do presente estudo, a pandemia causada pela COVID-19 afetou a vida social dos jovens e diminuiu o consumo de bebidas alcoólicas pelos adolescentes brasileiros, fenômeno associado a fatores sociodemográficos (idade e região de residência), adesão a medidas de restrição social, eventos relacionados à saúde mental (número de amigos, qualidade do sono e sentimentos de tristeza e irritabilidade) e estilo de vida (fumante ativo e passivo e comportamento sedentário).

Tais evidências indicam que as políticas públicas de promoção à saúde e à prevenção contra o consumo de bebidas alcoólicas devem ser articuladas, envolvendo gestores, educadores, família e sociedade em geral. Torna-se urgente envolver a sociedade no debate sobre o consumo de bebidas alcoólicas por adolescentes, estabelecendo condições para avançar na melhoria da legislação, com melhor regulação desde a oferta até a venda de bebidas alcoólicas, pensando-se em especial na proibição do marketing dessas substâncias, como acontece com os cigarros.
