# Supplementary material for: Erratum
Source: Rev Bras Epidemiol. 2024 Sep 30;27:e20230007erratum. doi: 10.1590/1980-549720230007.supl.1erratum (PMC11476863; doi:10.1590/1980-549720230007.supl.1erratum)
Supplement: Related texts

# Correction: Errata

**DOI:** 10.1590/1980-549720230007.supl.1.1erratum

No artigo "**O consumo de bebidas alcoólicas entre adolescentes durante a pandemia de COVID-19, ConVid Adolescentes — Pesquisa de Comportamentos**", DOI: https://doi.org/10.1590/1980-549720230007.supl.1.1, publicado no periódico Rev Bras Epidemiol. 2023; 26(Suppl 1): e230007.supl.1:

**Na página 1 foi incluído:**

EDITORA CIENTÍFICA: Márcia Furquim de Almeida http://orcid.org/0000-0003-0052-1888
